# Effects of Web-Based Education Program for Self-Urination Management of Patients with Spinal Cord Injuries in Community: Preliminary Study

**DOI:** 10.3390/healthcare13172088

**Published:** 2025-08-22

**Authors:** Hyeon Jeong Jin, Minkyung Gu, Nam Kyung Oh, Sohyune Sok

**Affiliations:** 1 Department of Nursing, Graduate School, Kyung Hee University, Seoul 02447, Republic of Korea; gimog@naver.com (H.J.J.); vitaminonk@hanmail.net (N.K.O.); 2 Department of Nursing, College of Health Science, Daejin University, Pocheon-si 11159, Republic of Korea; g-minkyung@hanmail.net; 3 College of Nursing Science, Kyung Hee University, Seoul 02447, Republic of Korea

**Keywords:** urination, education, compliance, efficacy, nursing

## Abstract

Background/Objective: Community-dwelling spinal cord injury patients have many difficulties and disabilities in self-care of urination based on pathological mechanisms. This study aimed to examine the effects of a web-based education program for self-urination management on knowledge and compliance of self-urination management, self-efficacy, and psychological well-being of patients with spinal cord injury in the community. Methods: A quasi-experimental study design using a non-equivalent control group was employed. There was a total of 36 participants, including 18 of each intervention group and control group. Web-based education program for self-urination management as an intervention was applied to intervention group duration four weeks. Measures included self-urination management knowledge, compliance of self-urination management, self-efficacy, and psychological well-being. The self-urination management knowledge, compliance of self-urination management, self-efficacy, and psychological well-being were significantly improved in the intervention group applied to web-based education program for self-urination management. Result: The results show that the web-based education program for self-urination management was effective in improving self-urination management knowledge, compliance of self-urination management, self-efficacy, and psychological well-being of patients with spinal cord injury in the community. Conclusions: Health professionals can utilize the web-based education program for self-urination management to help people with spinal cord injury live healthy, independent lives in the community.

## 1. Introduction

Spinal cord injuries occur in 250,000 to 500,000 people worldwide yearly, with approximately 2500 new cases occurring in South Korea [1]. Patients with spinal cord injuries experience difficulties due to bedsores and various complications in their cardiovascular, respiratory, and urinary systems, even after the acute phase of treatment and chronic rehabilitation [2]. Moreover, most patients with spinal cord injuries—over 80%—have problems regulating self-urination due to neurogenic bladder disease related to lower urinary tract disorders [2]. This increases the risk of urinary complications, such as urinary incontinence, bladder stones, hydronephrosis, and ureteral reflux, which interfere with the daily activities of patients with spinal cord injuries and may cause anxiety, avoidance, and even depression, a mental illness common in severe cases [3,4]. Eventually, it can have a holistic impact on the quality of life of patients with spinal cord injuries; hence, it is imperative to implement an educational program that can stably improve the ability of patients with spinal cord injuries to manage self-urination [5].

To continuously implement self-urination management for patients with spinal cord injuries, it is imperative to promote awareness of the disease and knowledge of proper self-urination management [6]. For patients with spinal cord injuries struggling with self-urination, intermittent cleaning management, sharing information on preventing urinary incontinence and urinary tract infections is more helpful [6,7]. In this context, self-urination management knowledge in various aspects, such as monitoring regular urine output, should be prepared first [6,7]. Ensuring that continuous practices necessary for self-urination management are carried out is also crucial. The most important causal prerequisite here is self-efficacy [8]. Self-efficacy actively helps patients with spinal cord injuries implement self-urination management, reducing the risk of secondary complications. In addition, self-efficacy in patients with spinal cord injuries leads to positive behavioral changes in self-urination management and provides motivation [9]. Therefore, an intervention advocating self-efficacy should specifically support patients with spinal cord injuries so that they can better understand their disease and acquire the correct knowledge and skills needed to implement self-urination management [10]. This way, they will be able to increase their value psychologically, be more aware of their physical and mental health, and change their social activities, which can help restore their psychological well-being [11,12].

Recently, studies on self-urination management interventions for patients with spinal cord injuries have mainly focused on self-urination and preference for self-urination catheter according to the degree of upper limb function of spinal cord injuries, focusing on self-urination management for patients with intermittent clean urination [13,14,15]. In line with this, studies on the occurrence of secondary complications and chronic diseases in patients with spinal cord injuries were conducted in South Korea. Studies on neurogenic bladder and intestinal rehabilitation after spinal cord injuries also exist. However, most studies were conducted more than seven years ago. As a result, recent studies related to nursing care in South Korea for self-urination management of patients with spinal cord injuries were lacking [16,17]. Moreover, although the education program for self-urination management provided in the early stages of rehabilitation of patients with spinal cord injuries in South Korea incorporated online video materials with details such as information for patients with spinal cord injuries and their families, guidelines for neurogenic bladder disease, and understanding of self-urination management, the references were produced a long time ago. Thus, there is a paucity of up-to-date data that can be effectively used as an evidence base for current situations.

Studies also indicate that patients with spinal cord injuries living in the local community have difficulty going out due to limited access to public transportation and environmental factors [18,19]. Since these patients with spinal cord injuries living in the local community may have significantly less access to hospital facilities and related centers, educational programs for preventive self-urination management are more urgently needed. Hence, developing and applying a web-based education program that can contextualize learning according to individual circumstances would be very useful for patients with spinal cord injuries living in the local community who are unable to manage self-urination due to mobility and accessibility difficulties [20]. The advantage of accessing necessary information anytime and anywhere, as well as learning according to one’s initiative, is also a strength of the web-based education program.

**Figure 1:**
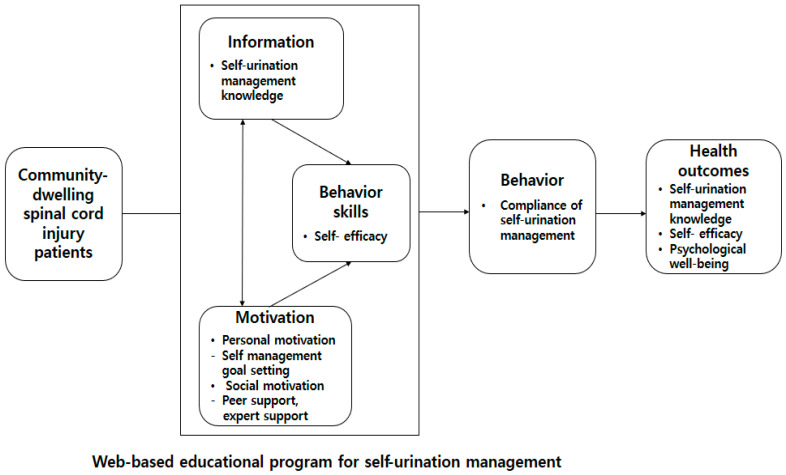
Conceptual framework of this study.

As for conceptual framework of this study, this study is based on the Information-Motivation-Behavior Skills (IMB) model proposed by Fisher and Fisher [21]. The IMB model presents information related to behavior change, motivation to induce behavior change, and behavioral skills based on information and motivation as prerequisites and constructs necessary for behavior change [22]. Here, the first construct (i.e., information) refers to the knowledge related to self-urination management required for patients with spinal cord injuries living in the local community to manage self-urination. In the second construct (i.e., motivation), personal motivation means having a positive attitude toward self-urination management by becoming more aware of the importance of self-urination management for setting self-urination management goals. The third construct refers to behavioral skills, which are self-urination management performance skills and self-efficacy. Through the online self-urination diary, patients can independently check their current self-urination volume and monitor their water intake. This section aims to strengthen the patients’ confidence and independence in self-urination management by helping them understand their condition better, enhancing their self-efficacy. Additionally, the program was designed to apply educational content to daily life through self-monitoring checks using educational materials, provided with information, phone calls, and social networking sites (Figure 1).

This study aimed to develop a web-based education program for self-urination management to provide health-related information to patients with spinal cord injuries living in the local community and examine its effectiveness.

## 2. Materials and Methods

### 2.1. Study Design and Participants

A quasi-experimental study design using a non-equivalent control group was employed (See Supplementary Materials [23]). The Korea Spinal Cord Injury Association was notified of the recruitment of study subjects. Subject candidates with spinal cord injuries living in the local community, recruited through voluntary participation, were selected. Specifically, patients who were discharged from the hospital after a spinal cord injury and are currently living in good health in the local community during the study period; were performing intermittent clean catheterization; were able to use the Internet and use mobile phones, tablets, or PCs; understand the study’s questionnaire; and respond voluntarily were asked to participate in the research. The subjects excluded from this study were minors under the age of 20, including those with visual, auditory, mental, and cognitive impairments. Those subjects who participated in similar educational programs within the last 6 months were also excluded to ensure the current study’s reliability of results. Participants were selected through convenience sampling, and they were randomly assigned to each group. If the coin flip resulted in heads, the subjects were assigned to the intervention group. The number of subjects was measured using the G-Power 3.1.9 program. The effect size was calculated based on the previous study of Jeon [24], who applied a smartphone application-based self-care program. The calculation of the significance level (α) of 0.05, power (1-β) of 0.80, and effect size of 0.60 indicated that the minimum sample size was 24 subjects, 12 in each group. Considering the 30% dropout rate, 36 subjects—18 in each group—were selected. However, there were no dropouts from the study.

### 2.2. Intervention

The web-based education program for self-urination management of patients with spinal cord injuries living in the local community was developed after completing five stages: analysis, design, development, operation, and assessment according to the web-based teaching-learning system design model of Jeong [25]. This model supplemented and modified the instructional design procedures required for the web-based teaching system design model (Table 1). A program information video on the contents and how to use the web-based education program for self-urination management was provided. Only the information of the intervention group subjects was registered as information of the education program site members. The first Monday of the week marked the beginning of the program participation when the subjects logged in and wrote the first self-urination diary. The educational program was applied through the website for four weeks, including eight sessions of video lectures (two sessions/week) and writing a daily self-urination diary. Regular SNS messages were used for continuous learning about self-urination management, and self-urination logs were created. Feedback on water intake and urine volume in the self-urination diary was also given periodically through phone calls once a week. If the study subjects have any questions or opinions, they could use the bulletin board, social media, and phone. The web-based education program for self-urination management in this study was designed from the design stage to allow for monitoring of participation indicators. Participants could select and attend individual lectures. Unattended sessions were marked with a red “Not Attended” indicator, while completed sessions were marked with a blue “Completed.” Furthermore, participants were encouraged to check their fluid intake and self-voiding volume goals during their self-voiding diary, which was designed to motivate participants. In terms of evaluation design, a separate administrator page was created to allow for bulk downloads of lecture attendance and self-voiding diary records. This allowed for verification of all participants’ course completion throughout the study period.

### 2.3. Measures

A set of general characteristic variables consisted of a total of 11 items including gender, age, family living together, monthly income, drinking, smoking, damaged area, duration of impairment, urinary incontinence, urine volume, and bladder medication.

The self-urination management knowledge section of the program was developed by the researchers based on the educational program content. The queries comprise a total of 46 questions including 14 questions on the structure and function of the bladder, nine questions on bladder activity after spinal cord injury, 13 questions on urination management, three questions on bladder treatment, five questions on bladder-related complications, and two questions on nursing care expense support. The measured score ranges from a minimum of 0 to a maximum of 46 points. The higher the total score, the higher the degree of knowledge related to self-urination. As regarding the self-urination management knowledge, the first and second content validity (Item-level content validity: I-CVI) tests were conducted on the questions developed. The experts were four nursing professors and two rehabilitation medicine physicians. The questions assessed how well the self-urination knowledge can be understood and measured on a four-point Likert scale. The first modified measurement tool was 80 for two items as a result of the second content validity. However, it was considered essential to measure self-urination management knowledge, so the content validity was set at 0.99 with 46 questions in total by deleting the two items. This study exhibited the reliability of the knowledge KR-20 (Kuder-Richardson Formula 20) for self-urination management at 0.75.

To assess compliance of self-urination management, we used a tool utilized by Wilde et al. [26] to measure the self-urination management for patients with spinal cord injuries. This tool includes 19 questions, such as “I drink enough fluids throughout the day”, “I pay attention to the color of urine, urine smell, and urine volume”, and “I am interested in the early symptoms of urinary tract infections”. The tool used a seven-point Likert scale where the score ranges from 19 points to 133 points, indicating that the higher the score of this tool, the better the self-urination management is implemented. Wilde et al. [26] presented that the reliability of the tool was Cronbach’s α = 0.82. The present study’s reliability of the tool was Cronbach’s α = 0.75.

To assess self-efficacy, we used a self-efficacy measurement tool utilized by Wilde et al. [26] for self-urination management for patients with spinal cord injuries. The tool features 12 questions, such as “I do what I want to do without being disturbed by other symptoms or health problems”, “I do what I want to do without being disturbed by physical discomfort or pain caused by the disease”, and “I try to reduce the number of visits to a doctor by doing various tasks and activities necessary for health care”. It is responded to using a 10-point Likert scale, with scores ranging from 12 points to 120 points, where the higher the score, the higher the self-efficacy for self-urination management. Wilde et al. [26] specified that the reliability of the tool was Cronbach’s α = 0.89. The current study’s tool reliability was Cronbach’s α = 0.84.

Psychological well-being was measured using a psychological well-being tool for self-urination management developed by Pinder et al. [27]. The tool comprises six questions, such as “I am aware of the need for self-urination”, “I sometimes feel embarrassed when I do self-urination”, and “I am worried about the risk of long-term complications of self-urination”. It uses a five-point Likert scale, with scores ranging from 6 points to 30 points, where the higher the score, the higher the psychological well-being for self-urination management. Pinder et al. [27] showed that the reliability of the tool was Cronbach’s α = 0.83. The current study’s instrument reliability was Cronbach’s α = 0.89.

### 2.4. Data Collection

**Figure 2:**
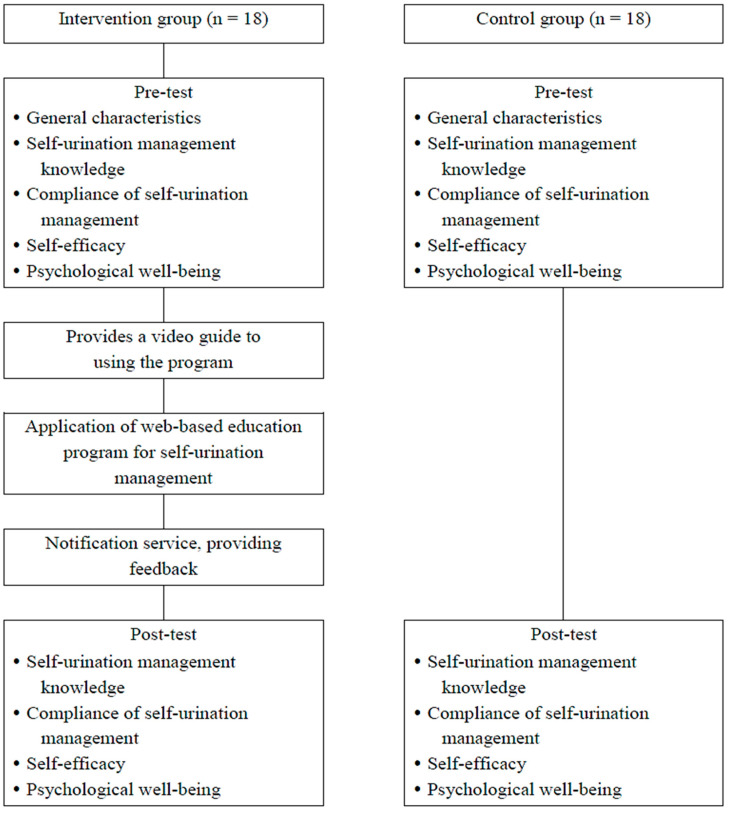
Flow chart of study process.

Data were collected from September 2021 to February 2022. The data collection period was set differently for the intervention group and the control group to prevent the occurrence of experimental diffusion. The experimental intervention was provided only to the intervention group. The data collection of the control group was first carried out using a staggered design to prevent disclosure of the program contents to the control group. The control group first conducted a preliminary survey, and a follow-up survey was performed four weeks after the preliminary survey. The preliminary survey of the intervention group was conducted after the data collection of the control group. The follow-up survey was administered immediately after the end of the four-week experimental intervention. This study did not disclose the randomly generated group numbers to the intervention group and the control group to prevent disclosing which group the subjects were assigned to. This study recruited 41 subjects. Five subjects who met the exclusion criteria were excluded from this study. Hence, 36 subjects were selected and analyzed in this study (Figure 2).

### 2.5. Ethical Considerations

This study was conducted after receiving approval from the Institutional Review Board in South Korea. First, the subjects were informed that they could withdraw their participation in the pre- and post-investigation and program at any time. The orientation includes the purpose and method of the study, the autonomy of participating in the research, the anonymity of participating in the research, and the possible benefits and disadvantages. Furthermore, the researcher explained that all personal information is anonymized and protected. Afterward, the subjects were asked to check whether they understood the contents of the research explanation and to fill out a consent form to participate in the study according to their own will. After the experiment was completed, all subjects were provided with a small gift certificate. The subjects selected as the control group were equally treated so that they could participate in the web-based education program for self-urination management after the study was completed.

### 2.6. Statistical Analysis

The collected data were analyzed using the SPSS/WIN 29.0 program. The general characteristics of study participants were analyzed using descriptive statistics as frequency, percentage, mean, and standard deviation. The homogeneity of the general characteristics and study variables at the pre-intervention between the intervention and control groups were analyzed using χ^2^-test and independent *t*-test. The normality of the study variables was calculated using Shapiro–Wilk. In order to examine the effects of the web-based education program for self-urination management, the pre-score was controlled and ANCOVA was used.

## 3. Results

### 3.1. General Characteristics of the Study Participants and Homogeneity

Table 2 displays the general characteristics of the study participants and the homogeneity test between the two groups. Homogeneity of the general characteristics between the two groups was secured by no statistically significant differences. In this study, the homogeneity of self-urination management knowledge (t = 1.68, *p* = 0.103), self-urination management compliance (t = 0.03, *p* = 0.980), self-efficacy (t = 0.08, *p* = 0.940), and psychological well-being (t = 0.87, *p* = 0.393) between the intervention and control groups before participating in the web-based education program showed that there were no statistically significant differences between the two groups, indicating homogeneity.

### 3.2. Effects of Web-Based Education Program for Self-Urination Management

Table 3 presents the effects of web-based education program for self-urination management. In this study, assumptions were verified before conducting ANCOVA. As a result, self-urinary management knowledge (t = 1.68, *p* = 0.103; F = 29.34, *p* < 0.001; F = 1.29, *p* = 0.309) and self-urinary management compliance (t = 0.03, *p* = 0.980; F = 62.91, *p* < 0.001; F = 2.01, *p* = 0.167) met the assumptions of prior homogeneity, linearity, and homogeneity of regression coefficients, but self-efficacy (t = 0.08, *p* = 0.940; F = 12.44, *p* = 0.004; F = 19.49, *p* < 0.001) and psychological well-being (t = 0.87, *p* = 0.393; F = 58.84, *p* < 0.001; F = 3.46, *p* = 0.036) did not meet the assumption of homogeneity of regression coefficients. To exclude the influence of differences in pre-test scores for all variables and to confirm the effects of a purely web-based educational program, an ANCOVA was conducted with pre-test scores as a covariate. Furthermore, to confirm the actual magnitude of the effect along with statistical significance, Cohen’s d was calculated based on the post-test scores. The analysis results showed significant effects or greater in self-care knowledge (d = 2.06), self-care compliance (d = 1.19), self-efficacy (d = 1.77), and psychological well-being (d = 2.12). Thus, statistically significant differences in self-urination management knowledge, self-urination management implementation, self-efficacy, and psychological well-being post-scores between the two groups according to participation in the web-based education program were found (Table 3).

## 4. Discussion

Regarding the general characteristics of this study, female patients with spinal cord injuries managed better self-urination than male patients. This finding supports another study’s claim that male patients with enlarged prostates are more likely to neglect self-urination management as they may feel uncomfortable expressing their urinary problems and are reluctant to disclose them [28]. Therefore, male patients with spinal cord injuries should be informed of the complications that may occur during treatment to debunk misconceptions centered on customized education programs. Also, to help them improve their self-urination management, providing specific feedback according to the clinical progress is necessary. This study revealed that for the intervention group that received a web-based education program for self-urination management, knowledge of self-urination management generally increased, compared to the control group. This finding substantiates the findings of Johns et al. [29] purporting that various interventions using in-person education, record-check logs, and telephone counseling feedback provided by medical staff are effective means of acquiring the medical knowledge necessary for self-care. Hence, constant guidance on rehabilitation preparation, adaptation, and strategies for managing chronic conditions should be provided, especially for recently diagnosed patients. Moreover, additional response support programs can help prevent relapse when returning to the local community.

This study demonstrated that the implementation of self-urination management was increased in the intervention group that received a web-based education program for self-urination management, compared to the control group. This outcome supports the research findings of Hussein and Mohammed [30] and Huang et al. [31], which postulated that continuous monitoring of the catheterization method was more positive in patients’ implementation of rehabilitation treatment. Ultimately, this result indicates that it is necessary to motivate patients with spinal cord injuries to actively participate in employing self-urination management by having them write self-urination diaries, etc. If self-urination is not properly carried out due to failure to recognize it as a risk factor, the side effects that occur to the urinary system of patients with spinal cord injuries are expected to be greater. Accordingly, monitoring of urination logs and catheterization methods will be useful in preventing recurrence and ensuring effective long-term management strategies for patients with spinal cord injuries. This study exhibited that the self-efficacy of the intervention group that received the web-based education program for self-urination management increased compared to the control group. This is in line with research showing that people with high self-efficacy tend to be more interested in their diseases and could take better care of themselves due to their consistent lifestyle [32]. Therefore, since self-efficacy can play an important role in the emotional well-being and therapeutic decisions of patients with spinal cord injuries, nurses who attend to patients with spinal cord injuries should consider helping them manage their urination on their own while increasing their self-efficacy.

In summary, the psychological well-being of the intervention group that received a web-based education program for self-urination management increased. This is consistent with the study’s results that patients with spinal cord injuries had an increased sense of psychological well-being after intermittent clean catheterization education [26]. The situation of having to manage self-urination care can encourage negative emotions, such as depression and withdrawal, in patients with spinal cord injuries. Therefore, it is critical to establish public health partnerships with governments in the local community to correct misinformation and actively integrate psychological factors in educational programs for patients with spinal cord injuries.

As for implications, the web-based education program for self-urination management in this study is a highly accessible program for patients with spinal cord injury who have limited mobility and will likely be highly utilized in the future. In addition, this study is significant in that it utilizes the direct and indirect support system of the community and is a more efficient education program for patients with spinal cord injury to lead an independent life related to self-urination management. Also, it will be very effective for managing urination habits through self-urination monitoring and continuous self-urination management for patients with spinal cord injury, not only in the early stage but also in chronic spinal cord injury. In the future, longitudinal studies are needed to verify the sustained over time effectiveness of education programs, including various clinical indicators such as urinary tract infection and bladder management tests for patients with spinal cord injury. Future studies need to strengthen internal and external validity by reflecting diverse cultural and social backgrounds and conducting multivariate analyses that consider potential factors. Qualitative studies are suggested to explore the experiential content of self-urination management education programs experienced by patients with spinal cord injury living in the community.

This study has several limitations. The study included only 36 participants (18 per group), which limits statistical generalizability. Also, this study applied a web-based education program for self-urination management to community-dwelling spinal cord injury patients with various injury sites, injury levels, and injury durations, so there are limitations in expanding or generalizing the study results. Generalization to other groups or environments is limited because it fails to include potential confounding variables such as socio-psychological factors, lifestyle habits, and environmental factors. The study only measured immediate post-intervention effects. No follow-up data are available to assess whether improvements in knowledge, compliance, or psychological well-being are sustained over time. In this study, there were no objective clinical markers, such as the frequency of urinary tract infections, episodes of incontinence, or bladder scan data, that would strengthen the clinical relevance. All outcome measures (knowledge, compliance, self-efficacy, and psychological well-being) were self-reported. These points may be limitations of this study. However, these limitations of this study can suggest directions for future research.

## 5. Conclusions

In conclusion, the web-based education program for self-urinary management was confirmed to be effective in improving the level of self-urinary management compliance in community-dwelling spinal cord injury patients, and in improving self-urinary management knowledge, self-efficacy, and psychological well-being. The web-based education program in this study is expected to enable self-urinary management in community-dwelling spinal cord injury patients, thereby improving their quality of life. It also intends to help prevent secondary complications caused by spinal cord injuries and increase the accessibility of educational programs for self-urination management of patients with spinal cord injuries without time and space constraints.

## Figures and Tables

**Table 1:** Web-based education program for self-urination management.

Menu	Content
Program information	Full program menu Program process and precautions
Self-urination management education	8-session video lectures -1st session: Structure and function of the urinary system -2nd session: Spinal cord injury -3rd session: Bladder after spinal cord injury -4th session: Urination management–general management -5th session: Urination management–urination method -6th session: Treatment -7th session: Urinary system complications -8th session: Nursing care expense support
Self-urination Logs	Submenu (Log entry) -Sample table of water content per food and beverage -Entry of self-urination diary (tabs, date, time, water intake, urine volume, urine color)
Bulletin board	Available for anyone to write on (experience sharing)
Q & A	Writing a 1:1 private inquiry to researchers

**Table 2:** General characteristics of study participants and homogeneity.

Characteristics	Intervention Group (*n* = 18)	Control Group (*n* = 18)	χ^2^ or t	*p*
	*n* (%)	*n* (%)		
Gender			5.60	0.081
Female	4 (22.2)	11 (61.1)		
Male	14 (77.8)	7 (38.9)		
Age (year)			0.56 †	0.757
21–40	6 (33.3)	5 (27.8)		
41–50	4 (22.2)	6 (33.3)		
51≤	8 (44.4)	7 (38.9)		
Family living together			0.11	0.735
Yes	11 (61.1)	10 (55.6)		
No	7 (38.9)	8 (44.4)		
Monthly income (Man won)			0.13	0.717
200>	12 (66.7)	13 (72.2)		
200≤	6 (33.3)	5 (27.8)		
Drinking			2.00	0.157
Yes	8 (44.4)	4 (22.2)		
No	10 (55.6)	14 (77.8)		
Smoking			0.23	0.630
Yes	3 (16.7)	2 (11.1)		
No	15 (83.3)	16 (88.9)		
Damaged area			1.14 †	0.775
Cervical spine	5 (27.8)	4 (22.2)		
Thoracic spine	12 (66.7)	11 (61.1)		
Lumbar spine	1 (5.6)	3 (16.7)		
Duration of impairment			1.18	0.471
Less than 60 months	7 (38.9)	4 (22.2)		
More than 60 months	11 (61.1)	14 (77.8)		
Urinary incontinence (frequency)			9.95 †	0.070
Every day	1 (5.6)	6 (33.3)		
Several times a week	3 (16.7)	3 (16.6)		
Several times a month	5 (27.8)	5 (27.8)		
More than a month	2 (11.1)	0 (0.0)		
several times				
None	7 (38.8)	4 (22.3)		
Urine volume (mL)			1.18 †	0.711
300>	6 (33.3)	6 (33.3)		
300–400	11 (61.1)	9 (50.0)		
400<	1 (5.6)	3 (16.7)		
Bladder medication			3.01	0.083
Yes	4 (22.2)	9 (50.0)		
No	14 (77.8)	9 (50.0)		

† Fisher’s exact test.

**Table 3:** Effects of web-based education program for self-urination management (*N* = 36).

Variables	Group	Pretest	Posttest	F †	*p*
		Mean ± SD	Mean ± SD		
Self-urination management knowledge	Intervention (*n* = 18)	25.28 ± 3.66	35.83 ± 3.97	48.04	<0.001 *
	Control (*n* = 18)	22.22 ± 6.80	25.00 ± 6.29		
Compliance of self- urination management	Intervention (*n* = 18)	92.94 ± 13.69	104.78 ± 6.39	45.86	<0.001 *
	Control (*n* = 18)	92.83 ± 13.16	92.88 ± 12.56		
Self-efficacy	Intervention (*n* = 18)	73.56 ± 9.82	91.72 ± 6.64	58.47	<0.001 *
	Control (*n* = 18)	73.22 ± 15.81	71.22 ± 14.97		
Psychological well-being	Intervention (*n* = 18)	15.33 ± 6.20	20.11 ± 3.03	98.86	<0.001 *
	Control *(n* = 18)	13.83 ± 3.96	13.11 ± 3.56		

† Result of ANCOVA controlling the values at pretest as a covariate, * *p* < 0.05.

## Data Availability

The original contributions presented in this study are included in the article. Further inquiries can be directed to the corresponding author.

## Supplementary Materials

The following supporting information can be downloaded at: https://www.mdpi.com/article/10.3390/healthcare13172088/s1, Table S1: TREND Statement Checklist [23].
